# Why do farmers and veterinarians not report all bovine abortions, as requested by the clinical brucellosis surveillance system in France?

**DOI:** 10.1186/1746-6148-10-93

**Published:** 2014-04-24

**Authors:** Anne Bronner, Viviane Hénaux, Nicolas Fortané, Pascal Hendrikx, Didier Calavas

**Affiliations:** 1 Anses-Lyon, Unité Epidémiologie, 31 avenue Tony Garnier, 69364 Lyon Cedex 07, France; 2 Unité RiTME, Inra, 65 Boulevard de Brandebourg, 94 200 Ivry-sur-Seine, France; 3 Anses, Unité de surveillance épidémiologique SURVEPI, Direction scientifique des laboratoires, 22 Rue Pierre Curie, 94700 Maisons-Alfort, France

## Abstract

**Background:**

Since 2005, France has been officially free of brucellosis, an infectious disease that causes abortion in cattle and can be transmitted from cattle to humans. Recent animal and human cases have drawn attention to the need to prevent infection of humans and animals from any primary outbreaks. In order to detect any new outbreaks as soon as possible, a clinical surveillance system requires farmers and veterinarians to report each abortion and to test the aborting cow for brucellosis. However, under-reporting limits the sensitivity of this system. Our objective was to identify the barriers and motivations influencing field actors in their decision to report or not to report bovine abortions. We used a qualitative approach with semi-structured interviews of 12 cattle farmers and their eight veterinarians.

**Results:**

Our analysis showed that four main themes influence the decision-making process of farmers and veterinarians: 1) the perceived risk of brucellosis and other abortive diseases; 2) the definition of a suspected case of brucellosis and other abortive diseases adopted by field actors, which is less sensitive than the mandatory definition; 3) the cost-benefit analysis conducted by actors, taking into account regulatory and health aspects, economic and financial losses, technical and practical factors; 4) the level of cooperation within the socio-technical network. We discussed how early detection may be improved by revising the definition of abortion, extending the time frame for notification and generalising the differential diagnosis of the causes of abortion.

**Conclusions:**

In contrast to quantitative approaches, qualitative studies can identify the factors (including unknown factors) influencing the decision-making process of field actors and reveal why they take those factors into consideration. Our qualitative study sheds light on the factors underlying the poor sensitivity of clinical brucellosis surveillance system for cattle in France, and suggests that early detection may be improved by considering actors’ perceptions. We believe our findings may provide further insight into ways of improving other clinical surveillance systems and thus reduce the risk of disease.

## Background

In the context of increasing cross-border movements of people and growing international trade of animals and animal products, the identification of health hazards before they emerge and spread is of utmost importance for both human and animal communities. From the late 1990s, the number of emerging and re-emerging infectious diseases has dramatically increased [1]. About 75% of the new diseases that have affected humans over the past ten years have been caused by zoonotic pathogens, i.e. pathogens that can be transmitted from animals (the main reservoir of the disease) to humans [2]. It is thus essential to detect any outbreak of a disease in animals as early as possible to prevent primary sources spreading the disease to other animals or humans. Clinical surveillance systems have therefore been implemented for several animal diseases, all of them relying on the mandatory notification of suspected clinical cases by farmers and veterinarians. However, under-reporting is regularly cited as one of the main limitations of these networks [3,4]. In order to improve the sensitivity of these surveillance systems, it is essential to understand the decision-making process of field actors, and identify the factors they perceive as incentives or barriers to reporting suspected cases.

Brucellosis in cattle is an infectious disease caused by *Brucella abortus* (and less frequently to *B. melitensis* and *B. suis*) which primarily affects the reproductive organs of infected animals. It can be transmitted to humans, causing a febrile syndrome and complications such as orchitis, endocarditis or arthritis. In France, the disease was eradicated in the bovine population in 2003 and the country has been declared officially free of the disease since 2005. However, there remains a chance that the disease will reoccur: in 2012, two bovine brucellosis outbreaks were detected [5]. The first, in a beef cattle herd, was due to the introduction of an infected animal from Belgium, another officially disease-free country. The second, in a dairy cattle herd, may have been infected by wildlife (unpublished results). This second outbreak led to a human case in a child who ate raw milk cheese produced from the infected herd. This human case was diagnosed three months before the disease was detected on the cattle farm [5,6].

These recent cases remind us that the early detection of primary cases and prevention of any bovine brucellosis outbreaks remain crucial for both public and animal health considerations. Once introduced into a cattle herd, abortion is not only the main clinical sign of the disease, but also its main source of dissemination, large quantities of bacteria being excreted in the foetus and uterine fluids [7]. The clinical surveillance system therefore relies on the mandatory notification of any abortions. According to national regulations, farmers have to call their sanitary veterinarian (mandated by veterinary services to carry out regulatory interventions such as vaccination or sample collection) in the event of a bovine abortion, defined by the French "Code Rural" as the expulsion of the foetus or calf, stillborn or dying within 48 hours of birth [8]. The sanitary veterinarian then has to report the abortion and take a blood sample from the aborting cow to test for *Brucella* spp. In practice, an abortion is reported to the veterinary services when the blood sample is sent to the departmental laboratory (a French department being an administrative and territorial unit with a mean surface area of 5,800 km^2^). The farm where a suspected case has been reported is not isolated and both the veterinarian’s visit and brucellosis analysis are financed by public funds. The failure to report a detected abortion is punishable by a fine of 1,500 euros [9]. However, in practice, it is extremely difficult to identify farmers and veterinarians not complying with regulations, so this sanction is never actually applied. Clinical surveillance of brucellosis is complemented by active surveillance, which consists of annual serological tests of each herd based on either bulk milk samples from dairy cattle or serum samples from 20% of beef cattle over 24 months old. However, its objective is more to prove the official disease-free status of France for bovine brucellosis than to ensure early detection [10].

Abortion is not a clinical sign specific to brucellosis. Several abortive endemic diseases such as Q fever, neosporosis and bovine viral diarrhoea may also cause abortions and thus direct economic losses for farmers. To help identify the cause of abortion, the GDS animal health groupings (*Groupements de Défense Sanitaire*, a departmental association of stock farmers addressing health issues, recognised in an official capacity under French law) for some departments have developed a differential abortion diagnosis protocol including alternative abortive diseases endemic to France. The GDS funds part of the analyses should the differential diagnosis protocol be followed.

However, despite national regulations and the importance to farmers of preventing health and animal risks related to brucellosis and other abortive diseases, the under-reporting of bovine abortion remains of major concern. In a previous study using capture-recapture methods, we found that the overall surveillance sensitivity, i.e. the proportion of farmers who reported abortion(s) out of all the farmers who had detected abortion(s), was about 20% for beef cattle herds and 39% for dairy cattle herds [3].

It is crucial to assess the willingness of farmers and veterinarians to participate in the system and the constraints that may influence their decision in order to identify the best ways of improving brucellosis risk management [11]. Yet, although effective and reliable surveillance requires motivated participants, there is still much research to be done on the social aspects of participation in animal disease surveillance systems [12]. Previous studies on the participation of these actors in clinical surveillance systems for avian influenza or scrapie in small ruminants have highlighted several potential barriers to reporting. These include a lack of knowledge and awareness of the disease; guilt, shame and prejudice; a negative opinion of control measures; dissatisfaction with post-reporting procedures; a lack of trust in veterinarians and government; a lack of transparency in reporting procedures, and finally, uncertainty about the notification process [13-15]. However, in our case, we hypothesise that these reasons do not fully explain the low abortion reporting rate because of the existence of precise criteria to define a suspected case and the absence of farm isolation after notification.

The objectives of this study were to understand farmers’ and veterinarians’ decision-making process when choosing whether or not to report abortions, and to analyse the role of differential diagnosis in this process. We used a qualitative approach with semi-structured interviews of field actors. Open questions were designed to encourage them to talk about their attitudes and perceptions [16]. We sought to identify the factors influencing their decision, potential interactions among those factors, and to contextualise the reporting or non-reporting decision in terms of multiple factors, such as institutional, social, psychological, technical or economic factors. Ultimately, these consultations with field actors were designed to reveal the barriers and motivations influencing the decision of farmers and veterinarians to participate in the clinical brucellosis surveillance system.

## Methods

### Study design

The study was conducted in two French departments named, for the purpose of this study, "A" and "B" and located in north-eastern and south-eastern France respectively. Department A hosted 1,674 cattle herds at the beginning of 2010 and the last bovine brucellosis outbreak was recorded in 1993. Department B hosted 4,177 cattle herds at the beginning of 2010 and the last bovine brucellosis outbreak was recorded in 2001. Taking into account production type and herd size, the proportion of farmers notifying abortions in department A did not differ significantly from the national level, whereas in department B, it was about 1.5 times higher than the national level. In both departments, the GDS animal health grouping provides financial support for differential diagnosis. In department B, a differential diagnosis protocol has to be followed for the farmer to benefit from this financial support.

### Pre-selection of participants

In each department, representatives of the veterinary services, GDS and GTV technical veterinarian association were interviewed separately to gather information about certain aspects of the implementation of the mandatory abortion notification system (including the roles of departmental veterinary services and the GDS, relationships between actors involved in surveillance and information provided by the GDS to farmers) and the differential diagnosis following abortion (for instance the existence of a protocol and extent of financial support). According to the "grounded theory" approach [17], participants were chosen purposively to include a variety of herd characteristics and different attitudes to having to report abortions: farmers were pre-selected taking into account the herd production type (dairy, beef or a mixture), the number of abortion(s) reported over the last two years, and participation in cattle performance recording programmes. In these programmes, farmers are subject to frequent checks on cattle performance, including milk production for dairy cattle, and maternal qualities or morphology for beef cattle. Committed to the long-term improvement of their herd, these farmers are assumed to have better farming practices and thus be more prone to reporting abortions than other farmers.

The GDS contacted potential participants by phone to provide information on the purpose and nature of the study. One farmer wished not to be interviewed due to lack of time and one veterinarian refused categorically to participate in the study (and did not explain why). Participants who agreed to be interviewed were then contacted by phone by the person in charge of the study. The aim, nature and background of the study were explained in detail, and potential participants were informed that their data would remain anonymous and that any material potentially leading to individual identification would be removed. Once verbal consent was obtained, a time was arranged for the survey to be conducted. Farmers and veterinarians had no financial incentive for participating in the study.

### Data collection

In-depth interviews were conducted by the same person at the participants’ location of choice (office or home) from October to December 2012. At the beginning of the interview, the aim and background of the study were recalled, as was the confidentiality of the interview. These issues were presented in a document that was given to each participant. In accordance with [18], participants were again asked to provide verbal informed consent prior to the interview. It was made clear that by agreeing to be interviewed, they were agreeing to be part of the study. All participants agreed to the interview being recorded.

Interviews lasted between 50 and 105 minutes. Through open-ended questions, the interviewer asked the farmer or veterinarian to talk about their knowledge of abortive diseases, their perception of this issue, and the difficulties and barriers to participating in the surveillance system (Table 1). In all, 12 farmers and their eight sanitary veterinarians were involved in the study; a sample size justified by interviewing participants until "theoretical saturation" [17] was achieved (i.e. no novel idea was raised during the most recent interviews). For all but one, the farmers’ sanitary veterinarian was the same as their private veterinarian practitioner who treated their animals. The characteristics of the interviewed farmers are provided in Table 2.

**Table 1:** Topics of discussion during farmer and veterinarian in-depth interviews

**Participants**	**Topics of discussion**
Farmers	Number of abortions detected in the last two years, circumstances of detection
Veterinarians	Circumstances of a farmer’s call in the event of abortion(s)
Farmers and veterinarians	Definition of abortion
	Measures taken in the event of abortion and reasons
	Knowledge about the mandatory abortion notification system, and the differential diagnosis protocol and/or financial and technical support by the GDS
	Type of information obtained about bovine abortions, the mandatory bovine abortion notification system and differential diagnosis actions
	Expectations about abortion surveillance actions or information

**Table 2:** Characteristics of farmers

**Production type**^**1**^	**Start of the farmer’s activity**	**Number of breeding cattle**	**Number of reported abortions (detected abortions**^**2**^**)**		**Department**
			**in 2011**	**in 2012**	
Dairy	2005	70-80	0 (3)	1 (1)	A
Dairy*	1990	130	5 (5)	4 (4)	A
Dairy*	1990	130-150	1 (9)	2 (2)	A
Dairy*	2008	65	0 (1)	0 (0)	A
Mixed	1992	65	1 (1)	0 (4)	A
Mixed*	1997	270	0 (about 14)	0 (about 14)	A
Mixed	1992	30-40	0 (5)	0 (2)	A
Mixed*	1991	110	0 (2)	0 (5 to 8)	A
Mixed*	2003	160	2 (3–4)	0 (1)	B
Beef*	1993	65-70	0 (1)	0 (0)	B
Beef*	2006	50	0 (0)	7 (7)	B
Beef	2001	85-90	0 (1)	1 (1)	B

^1^Herds included beef cattle, dairy cattle, or a mixture of both types of production.

^2^The number of detected abortions was collected during the interviews with farmers.

*Farmers participating in the cattle performance recording programme.

### Data analysis

The interview notes were transcribed from the audio recording and analysed using thematic analysis [19]. They were reviewed and a code given to each key word or sentence. Similar codes were grouped into categories, and categories were gathered into sub-themes and themes [19]. The credibility and rigor of the analysis were aided by co-analysis of transcripts by two of the researchers and continual re-examination of the emergent data throughout the research process. Discrepancies in interpretation were discussed and resolved during consensus meetings. All the data presented in the results section reflect the observations, insights and opinions expressed by participants. In addition, a typology of the interviewed farmers was constructed to study the main factors driving their decision-making process based on two considerations: their perception of risk and their attitude towards abortion notification. They were scored on these two considerations as negative or positive, i.e. low versus high perceived risk of introducing an animal disease into their herd for the first consideration, and low versus high rate of notification of detected abortions for the second consideration.

Data collection and analysis were conducted in accordance with the research ethics requirements of the American Sociological Association [18] and qualitative research review guidelines (Additional file 1).

## Results

### Factors influencing farmers’ and veterinarians’ decision-making process

We defined health risk as the likelihood of the occurrence of a negative health event for people or animals. Four main themes emerged from the analysis of farmers’ and veterinarians’ interviews: their perception of the risk of brucellosis and other abortive diseases; the definition of a suspected case related to the risk of brucellosis and other abortive diseases; cost-benefit analysis and socio-technical factors.

#### Theme 1: Risk perception of brucellosis and other causes of abortion

Brucellosis was perceived by most farmers and veterinarians as a serious disease, particularly when they had experienced brucellosis in the past. However, the probability of an outbreak occurring was usually perceived as negligible, i.e. most farmers did not fear a potential introduction of brucellosis when an abortion occurred in their herd. The detection of two brucellosis cattle outbreaks and one human case in France in 2012 did not change their perception of this risk because these cases were distant (i.e. not in a neighbouring department). In contrast, some farmers were conscious of the potential role of wildlife in the transmission of diseases (e.g. tuberculosis) to cattle and feared a brucellosis infection of their cattle herd by wild animals, or the introduction of an endemic disease such as bovine viral diarrhoea by contact with an infected neighbouring cattle herd. In these cases, farmers usually requested a test for brucellosis or another disease.

#### Theme 2: Definition of a suspected case related to the risk of brucellosis and other abortive diseases

**Figure 1:**
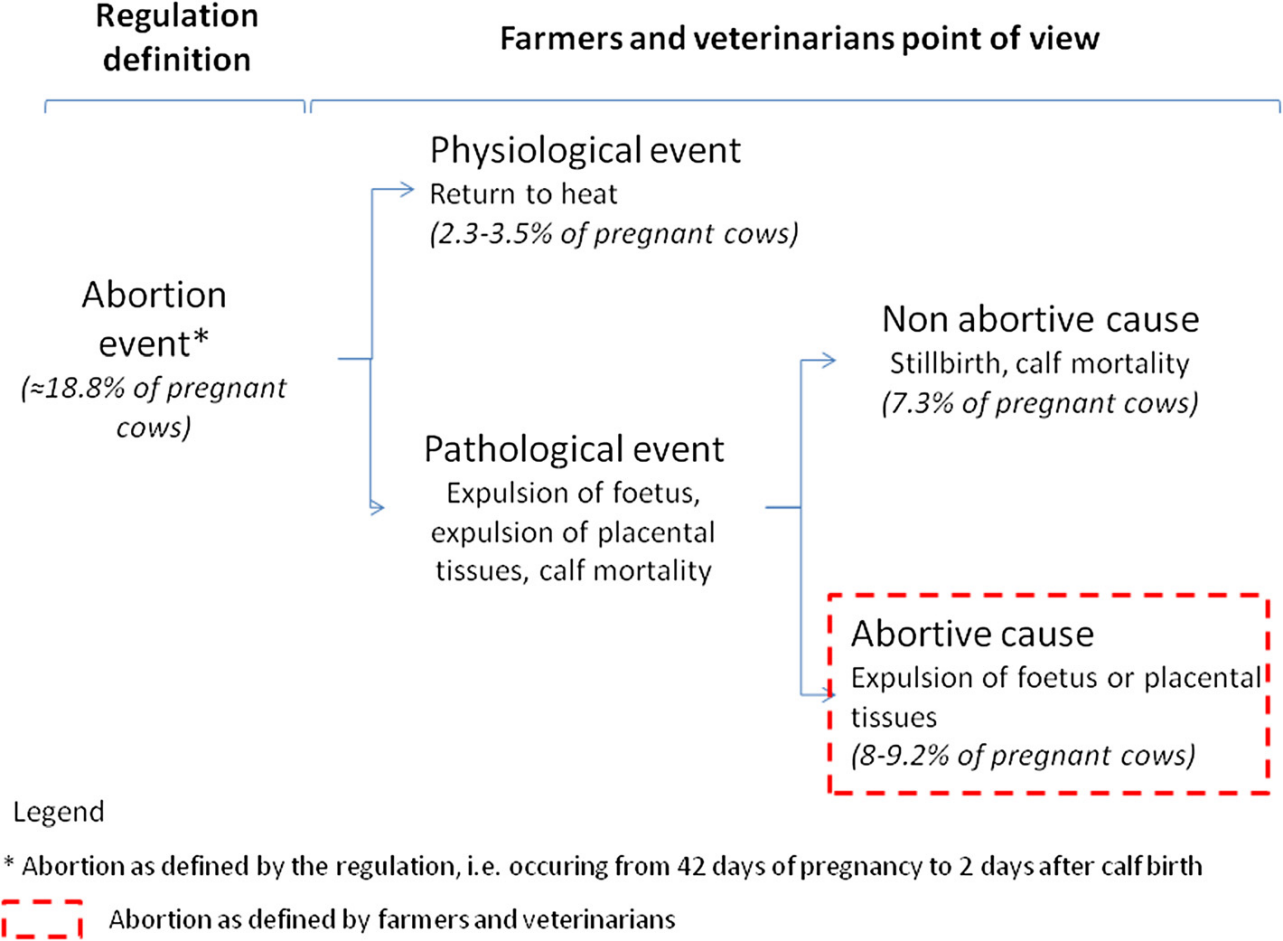
**Difference between the official definition of abortion and the definition of farmers and veterinarians.** Abortion is defined by French regulations as an interruption of pregnancy occurring from 42 days of pregnancy to term, or as the death of a calf within 48 hours of its birth. A recent study of the time between artificial insemination and calving in dairy cattle estimated that the rates of abortion occurring in mid-pregnancy and late pregnancy were about 6.4% and 5.1% respectively [20]. As only 20 to 30% of abortions are detected visually [21], only 70% to 80% of these aborting cows are detected by field actors, i.e. 8 to 9.2% of pregnant cows. Furthermore, the 7.3% or so of calves that die within 48 hours after birth are supposed to be systematically detected [22].

The interviews revealed that the word "abortion" had a negative connotation. Farmers and veterinarians defined an abortion as the direct observation of a foetus or placental tissues that had just been expelled from the cow, which is a more restrictive definition than the official one (Figure 1). Although a cow returning to heat more than 42 days after an attempt at fertilisation may be a sign of an interruption of pregnancy, farmers and veterinarians did not usually consider this event as the consequence of an abortion, arguing that unsuccessful mating causes returns to heat that may be detected late (more than 42 days after an attempt at fertilisation). Thus, as most of the abortions occurring before 5–6 months of pregnancy or those in beef cows at pasture are usually detected by a return to heat, these events were not reported. Furthermore, farmers considered that abortion was the fault of the cow, whether due to an abortive disease or other cause. Stillbirths or death of the calves soon after birth, which are also officially considered abortions, were not reported because they were believed to result from calving difficulties or calf illness rather than from an abortive disease.

Abortion (as defined by farmers and veterinarians) was considered a normal event as long as it remained sporadic and under a "threshold" proportion in the herd, which ranged among interviewed farmers from 1.5 to 5% a year. Most farmers were confident in diagnosing abortions themselves, with common causes including accidents or feed-, medication- or health-related issues. Therefore, they did not feel the need to consult their veterinarian in the event of a sporadic abortion or if a non-infectious cause was suspected. All the farmers and even some veterinarians were more prone to carry out biological analyses in the event of recurrent abortions: one veterinarian was not in the habit of reporting abortion in the case of a return to heat, but mentioned reporting abortions on a farm where more than half of a group of heifers had returned to heat.

#### Theme 3: Cost-benefit analysis

Farmers and veterinarians weighed the benefits and costs of both reporting abortions and conducting a differential diagnosis. Multiple factors, including regulations, health, economic, financial, technical, and practical considerations, were taken into account in that process (Tables 3 and 4). The role of regulations appears to be mitigated by the absence of sanctions and the perceived lack of relevant measures to ensure early detection of brucellosis. Farmers and veterinarians did not report abortions in order to meet the objective of the brucellosis surveillance system—detecting an outbreak as soon as possible—but to comply with their direct preoccupations. They were more concerned about their animals’ health (for economic and professional reasons) than about public health issues.

**Table 3:** Cost-benefit analysis by farmers deciding whether to call their veterinarian for an abortion

**Factors**	**Benefits**	**Costs**
Regulations	Professional conscientiousness	No added-value for the farmer as there is no enforcement
	Avoid sanctions	
		Lack of technical justification: they believed a brucellosis outbreak would be detected by a significant abortion episode or by active surveillance
Health	Identify cause of abortion	Difficulties in identifying the cause: one farmer stopped reporting abortions after an unsuccessful differential diagnosis to identify the cause
	Ensure the absence of a specific disease or diseases in general (some farmers were not aware that brucellosis is the only disease tested)	
	Care for the aborting cow	
Financial	Free visit	Financial costs of additional analyses and sanitary/medical measures to prevent further abortions
Economic	Prevent further abortions	Lower sales of animals from a herd with seropositive animals
Practical		Animal has to be caught
		Time-consuming

**Table 4:** Cost-benefit analysis by veterinarians deciding whether to report abortions and make a differential diagnosis

**Factors**	**Benefits**	**Costs**
Regulations	Professional conscientiousness	Lack of technical justification: a brucellosis outbreak would be detected by a significant abortion episode or by active surveillance
	Technical justification: brucellosis may cause late abortion (after six months of pregnancy	
Technical	Technical interest in identifying the cause of the abortion	Difficulties in identifying the cause of abortion
		Lack of knowledge: when veterinarians carried out a differential diagnosis, they included known abortive diseases with a known diagnosis protocol and effective measures to reduce the occurrence of abortions; their diagnosis protocol sometimes differed from scientific requirements. One veterinarian did not report an abortion because he did not know which diseases other than brucellosis to include
		Low impact of sanitary and medical measures to prevent further abortions due to enzootic diseases
Financial		The farmer refused to pay additional costs for analyses and sanitary/medical measures to prevent further abortions
Practical		Time schedule with farmer
		Lack of time to seek advice about differential diagnosis

The possibility of identifying the cause of abortion through a differential diagnosis motivated their decision to report. However, technical difficulties such as a lack of training, no standard differential diagnosis protocol, and absence of the placenta or foetal material make identifying the cause harder and thus discouraged some farmers and veterinarians from reporting abortions. Therefore, some veterinarians conduct a differential diagnosis test only if the placenta or foetal material is available. Furthermore, veterinarians pointed out the cost to farmers of additional analyses as a barrier to evaluating other abortive diseases, though most farmers mentioned that financial factors did not greatly influence their decision when they were worried about the occurrence of abortions. Nevertheless, their cost-benefit analysis varied with the herd production type: beef cattle farmers mentioned the practical difficulties in catching a cow at pasture for a serological analysis.

#### Theme 4: Socio-technical network

As sanitary veterinarians are mandated by French authorities, they were more prone to apply the mandatory measures than farmers. However, they underlined the difficulty of convincing reluctant farmers to report abortions. Furthermore, some veterinarians were positive about their relationships with veterinary services, others criticised the absence of technical support should they have difficulties in identifying the cause of abortion, and the lack of information about surveillance results (Table 5). The decision to report an abortion and carry out the differential diagnosis protocol was either the result of a consensus between the farmer and his veterinarian, or the initiative of the veterinarian. However, in the case of abortions, most farmers did not know if a differential diagnosis test was conducted or which diseases were included. Because of the possible negative consequences of a positive result for the farmer and to preserve their relationship with their clients, veterinarians took farmers’ expectations and difficulties into consideration. This was to the detriment of their obligation to report abortion, their financial interest in participating in the surveillance system, and their technical interest in conducting differential diagnosis.

**Table 5:** Socio-technical factors taken into account by farmers and veterinarians in their decision-making process

**Factor**		**Reasons for reporting abortions**^**1**^	**Reasons for not reporting abortions**^**1**^
Farmer	Relationships with veterinary services and the GDS		Limited interactions
			Farmers did not feel responsible for early detection of brucellosis
	Relationships with veterinarians	Trust in the veterinarian’s expertise (even though one farmer required further advice from the GDS)	No trust in the expertise of the sanitary veterinarian and consultation of another practitioner in the event of health problems
		Explanations by the veterinarian of the advantages and limits of differential diagnosis	Difficulties due to the absence of consideration by veterinarians: for example, animal sales had been stopped for several weeks after a seropositive result obtained from a differential diagnosis about which the farmer had not been informed
Veterinarians	Relationships with veterinary services and the GDS	Role of the sanitary veterinarian	Absence of a technical added-value
			Feeling of being under the supervision of veterinary services and the GDS
			Dissatisfaction with veterinary services including lack of information on surveillance results, lack of technical training, lack of discussion about their difficulties
	Technical network	Some veterinarians have their own expert network	Lack of technical support should they have difficulties in identifying the cause of abortion
	Relationships with farmers		Blame farmers for not systematically consulting them in the event of abortion despite their messages to increase farmers’ awareness
			Farmers’ expectations and difficulties taken into account: no differential diagnoses were performed on farms where animals were sold abroad or if technical difficulties in determining the cause of abortion were feared

^1^For farmers, reasons for calling their veterinarian in the event of abortions; for veterinarians, reasons for reporting abortion and performing a differential diagnosis.

### Typology of farmers

The classification of farmers according to risk perception and decision on abortion notification underlined the influence of regulation incentives, health incentives, practical difficulties, and poor integration in socio-technical networks (due to a lack of communication with their sanitary veterinarian and the GDS (Table 6). The perceived risk of brucellosis occurrence did not influence farmers’ decision on abortion notification in a deterministic manner. Farmers who perceived the risk of introducing brucellosis or another enzootic abortive disease into their herd as low reported abortions to comply with the law except when practical issues predominated. In the latter case, one farmer suggested the possibility of reporting abortions only if a disease was detected in his neighbourhood. In contrast, farmers who perceived the risk of introducing a disease into their herd as high did not consider abortion as a banal event and were better informed about diseases than the others (from GDS leaflets, information on the Internet or meetings organised by their veterinarian or GDS). However, some of them did not notify all abortions due a lack of trust in the sanitary veterinarian due to previous inconclusive diagnosis and expensive veterinary visits.

**Table 6:** Main factors driving the decision process of farmers according to risk perception and abortion notifications

		**Rate of abortion notifications**	
		**Low**	**High**
**Perceived risk of introduction of an animal disease in their herd**	**Low**	Practical difficulties	Respect for the law
	**High**	Lack of relationship	Sanitary and economical factors

The typology of interviewed farmers was based on two considerations: their perception of risk and their attitude towards abortion notification. They were scored on these two considerations as negative or positive, i.e. low versus high perceived risk of introducing an animal disease into their herd, and low versus high rate of abortion notification. On the basis of these two considerations, four groups of farmers were identified, being especially influenced by incentives, health and economic factors, practical difficulties, or poor integration in socio-technical networks respectively.

## Discussion

The low sensitivity of the mandatory notification system for bovine abortion limits the early detection of any potential introduction of brucellosis or other abortive diseases into France [3], with potentially important animal and health risks. In that context, our qualitative study investigated the barriers and difficulties that hinder the notification of a suspected case, and possible solutions or incentives to encourage farmers’ and veterinarians’ participation in brucellosis risk prevention. Although our study focused on the sensitive topic of fulfilling mandatory requirements, the confidentiality of the interviews warranted the trustworthiness of participants’ answers [16], as suggested by farmers admitting that they report none or only some of the detected abortions (Table 2). A review of data on abortion notifications enabled us to validate farmers’ assertions about their participation in the surveillance system.

### Specificity of the qualitative approach

In contrast to a quantitative approach, commonly used in epidemiology to estimate the proportion of actors influenced by specific predetermined factors, the goals of our study were to identify which factors (including unknown factors) influence their decision and to understand why those factors are taken into consideration by field actors [23]. The qualitative approach relies on "purposive sampling" to maximise diversity [17] by covering a broad spectrum of experiences and positions relative to a given phenomenon [24]. In our study, we selected a wide range of participants with different herd characteristics and a variety of attitudes towards their duty to report abortions. We found that "theoretical saturation" was reached after ten interviews for farmers and six interviews for veterinarians, which means that no new information was raised in the last interviews [17]. This finding is in agreement with other studies, for which saturation occurred within the first 12 interviews [25].

Purposive sampling and theoretical saturation ensured the robustness and trustworthiness of our study and enabled us to generalise the information provided by study participants [24] into four themes that may be used to interpret the decision-making process of other farmers and veterinarians or decision-making process concerning other clinical surveillance systems such as classical swine fever or foot and mouth disease [26]. In addition, within the four themes, factors influencing the decision-making process may be transferable to other related topics [26], as suggested by the similarity between our findings and concepts developed in other studies on notification decisions or behaviour regarding vaccination [27,28].

### Farmers’ and veterinarians’ decision-making process for reporting abortions

The typology of farmers according to their risk perception and attitudes towards the abortion notification process underlines little diversity in the driving factors once these two considerations are taken into account [14,29,30]. Furthermore, the same themes appear for both farmers and veterinarians. This result suggests that despite profession-related differences (interests, knowledge, position in the surveillance network), these actors share common interests in participating in the surveillance system. This contrasts with their divergent views on other issues such as the implementation of vaccination and culling strategies to control animal epidemics, where farmers have more to lose than veterinarians [31].

The decision of farmers and veterinarians to report or not to report abortions is driven by three main considerations: the perceived risk of brucellosis (or other abortive diseases); an evaluation of the individual benefits and costs linked to the abortion notification and differential diagnosis; the socio-technical network to which the farmers and veterinarians belong. The first two concepts—risk perception and cost-benefit analysis—are the backbone of the Health Belief Model (HBM), which is a framework widely used to describe health-related actions [32].

Risk perception is essentially based on lay knowledge and "value and belief-oriented rationality" [33], especially for farmers who rely on their experience and regular observations of their cattle herd. We also noticed the role of experience for veterinarians, although their judgement depends primarily on their technical and scientific knowledge. In contrast to risk perception, cost-benefit analysis is essentially based on "purposive and instrumental rationality" [33]. Farmers and veterinarians considered calculated reasons (including regulations and health aspects, economic and financial losses, technical and practical factors) and their own self-interest to guide their decision. The importance of some factors and thus the outcome of the cost-benefit analysis varies with the type of production. Indeed, the technical features of beef production compared to dairy cattle [34] reduce beef cattle farmers’ *margin for manoeuvre,* as they are bound by both practical and financial constraints. The grazing season increases their difficulties in detecting abortions and carrying out a serological analysis on cows, as they do not have the daily contact that dairy cattle farmers do with their cows. Moreover, in the event of an abortion, the cow is usually immediately culled and sent to the slaughterhouse, and farmers do not feel the need to notify the abortion.

Besides risk perception and cost-benefit analysis, there is a third consideration. The French veterinary services, GDS, GTV, sanitary veterinarians and farmers are all linked through a social and technical network [35]. The mandatory abortion notification surveillance system defines what should be done by each actor to facilitate abortion notification and ensure early detection of a brucellosis outbreak. However, even if farmers are theoretically best placed to report abortions and benefit the most from the surveillance system, it does not make sense for most of them to be required to report abortion as a public duty. This reluctance to report abortions may be compared to the reluctance of humans to vaccinate themselves [28]: in both cases, the decision-making process of most people is not driven by the need to fulfil a public duty but by self-interest. Moreover, some farmers decide not to report an abortion because other farmers do not (peer influence), considering they have performed the necessary cost-benefit analysis to make a wise choice [27,36].

### Perception of brucellosis risk and preventive measures

Like most clinical surveillance systems, the mandatory abortion notification system gives priority to sensitivity rather than specificity, given the sensitive definition of a suspected case. However, farmers and veterinarians do not feel the need to report every abortion to detect a brucellosis outbreak early, as most of them perceive the risk of brucellosis as low. The epidemiological situation regarding brucellosis in France has dramatically changed since the implementation of the surveillance system in 1965, when brucellosis was enzootic. Today, in a brucellosis-free context, farmers are more concerned about abortive enzootic diseases such as bovine viral diarrhoea or Q fever than brucellosis. Furthermore, as it has been cited elsewhere [37], high levels of confidence reduce perceived risks. In our case, farmers feel confident in their ability to detect a brucellosis case in their herd or in the effectiveness of the annual serological surveillance system for dairy and beef cattle herds. Abortion is a non-specific clinical sign that may or may not be caused by an infectious disease. Thus, reporting the abortion, illness, or death of a single animal does not make sense to them if the objective is to detect an infectious disease [13].

These pragmatic feelings are supported to some extent by the results of a simulation study which showed that abortion notification (with a reporting rate varying from 20% to 80%) tends to be a less effective method for the early detection of brucellosis than annual bulk milk surveillance [38]. However, some infected animals may not be identified by serological tests as infected cows may be serologically negative for several months until they give birth to a calf or abort [7,39]. Moreover, brucellosis infection may spread slowly, depending on how the disease is introduced into the herd (from an infected animal or by indirect transmission) and herd management factors, which may influence the amount of contact the animals have with each other, such that only a few animals in the herd may become seropositive or abort, even though brucellosis is a contagious disease [5,40].

### Prospects for improving brucellosis risk prevention

The mandatory notification of each abortion is viewed by most farmers as an externally imposed tool for an externally imposed issue (the need to detect a brucellosis outbreak early) that they are not actually worried about [41]. However, the multiple patterns of brucellosis transmission and dissemination underline the importance of monitoring abortions. In addition, this system is useful for the surveillance of other diseases causing bovine abortion such as Q fever or Rift Valley Fever. Efforts by veterinary services and the GDS to increase farmers’ and veterinarians’ awareness about the need to report abortions has not reduced the probability of under-reporting, which has remained stable since 2009 [3]. As underlined by psychologists, messages to change people’s attitude require three factors: a credible communicator, a high level of "similarity" between the audience and communicator, and finally both the message and communicator must be perceived as trustworthy [42,43]. The "traditional" communication strategy is based on rational arguments, but requires the actors’ basic motivation and an interest in the topic [44]. Our findings suggest that the difficulties in increasing abortion notification rates may be related to the low level of trust in the communicators (veterinary services and GDS) and the low level of concern about abortions as long as they remain sporadic. Moreover, we found that both farmers and veterinarians do not agree with the decision to report every single abortion, which they consider irrelevant and of no real use [45]. Thus, enhancing risk communication requires not only relying on the "rational choice" model of decision but also taking into account the actors' social context as well as their values, beliefs, and how much they trust the different sources of information [46].

Our analysis suggests that putting a great deal of effort into increasing the number of reported abortions without updating the surveillance procedure would be inefficient. First, brucellosis infection causes mid-term or late abortions, which occur between five months and the end of pregnancy [7]. Second, the current situation regarding brucellosis in France suggests that the human and financial resources required to meet national and international regulations, i.e. testing about 18.8% of cows (which suffered mid-term or late abortions, or had a calf that died within 48 hours of birth, [20,47]), seem disproportionate in comparison with the number of brucellosis outbreaks that would be detected promptly (one outbreak in 2003 and two cases in 2012). Third, the performance of such a system will always rely on farmers’ and veterinarians’ willingness to participate in it, and these field actors are not prone to report cows returning to heat or the death of newborn calves. Therefore, there is a real need to revise the surveillance procedure so that the system is more efficient. Considering OIE requirements [48], these changes need to be considered on an international scale. An initial suggestion would be to revise the definition of abortion by excluding stillborn and newborn calves. Furthermore, a brucellosis analysis could be requested only beyond a certain frequency threshold of abortions within the herd. Farmers could be requested to register each abortion themselves (in their own record or in a national record system) but call their veterinarian only in the event of recurrent abortions (for example, two abortions or more within a month). Besides, cows suspected to have aborted could be sampled after a certain delay should the abortion not be detected early enough, or if practical issues hinder the immediate sampling of the cow.

By helping farmers and veterinarians identify the cause of abortion, the differential diagnosis with respect to other abortive enzootic diseases was supposed to improve their willingness to contribute to the surveillance system and participate in brucellosis risk prevention. However, it has been estimated that the cause was only diagnosed for about one third of the biological submissions [49]. The difficulties in identifying the abortive pathogen arise from the wide range of potential pathogens, the ubiquity of pathogens such as Q fever or salmonellosis, and veterinarians’ lack of knowledge about the type of samples to collect for the analyses. Therefore, a differential diagnosis protocol has been recently drawn up nationally in order to help veterinarians with the sampling process and identification of the cause of the abortion. Improving the coordination of veterinarians by official bodies providing technical support, training and information on the results of the differential diagnosis protocol (in addition to the results from the mandatory surveillance system) is also expected to increase participation in the surveillance system [12].

These prospects for improving brucellosis risk prevention could be useful for improving other clinical surveillance systems. Indeed, while some factors related to the reporting bias are specific to brucellosis or cattle production, most of them may influence the decision-making process for other diseases. Many exotic diseases with clinical surveillance are seen as low-risk, and the decision-making process is likely to be driven by regulations, health, economic, financial and technical factors. Thus, we expect that farmers and veterinarians would be more likely to report avian influenza or classical swine fever suspicions in the event of high mortality or morbidity than if only a few animals fall ill or die (which is the case if the outbreak is due to low pathogenic AI virus or pestivirus) [14]. Enhancing risk communication, developing differential diagnosis and revising the surveillance procedure to find the best compromise between sensitivity, expenditure and acceptability are ways of improving clinical surveillance systems that should be taken into consideration.

Nonetheless, performance will always rely on farmers’ and veterinarians’ participation, and consequently under-reporting will remain the major limitation of such systems. In this context, it might be useful to develop other surveillance procedures, such as syndromic surveillance. As regards brucellosis, it might be useful to develop an indicator to identify the occurrence of abortions in cattle by using information from additional sources, such as the dates of artificial insemination or calving intervals.

## Conclusions

According to the U.S. Centres for Disease Control and Prevention, the acceptability of public health surveillance systems by participants is one of the attributes to be assessed when evaluating a surveillance system [11]. To our knowledge, our study is the first to investigate the factors underlying the participation of farmers and veterinarians in a mandatory clinical surveillance system where there is no farm isolation in the event of notification of a suspected case; this issue is one of the main barriers to reporting in other passive surveillance systems [14]. Our qualitative study sheds light on the factors underlying the high proportion of under-reporting farmers, and differences in the reporting rates between dairy and beef cattle farmers [3]. Several recommendations, including revising the definition of a suspected case, extending the time frame for notification, and providing adequate diagnostic tools, support and information to field actors, may improve their participation in the surveillance system. We believe these incentives and measures should also be considered in other clinical surveillance systems to improve the rate of notification of suspected cases, facilitate the detection of emerging pathogens, and improve animal and public health risk prevention.

## Competing interests

The authors declare that they have no competing interests.

## Authors’ contributions

AB performed the study and drafted the manuscript. NF, DC and PH participated in the conception, design and analysis of the study. VH contributed to the analysis and helped draft the manuscript. All the authors read and approved the final manuscript.

## Supplementary Material

Additional file 1 RATS checklist. Click here for file
