# Supplementary material for: Why do farmers and veterinarians not report all bovine abortions, as requested by the clinical brucellosis surveillance system in France?
Source: BMC Vet Res. 2014 Apr 24;10:93. doi: 10.1186/1746-6148-10-93 (PMC4036594; doi:10.1186/1746-6148-10-93)
Supplement: Additional file 1 — RATS checklist. [file 1746-6148-10-93-S1.doc]

## Additional file 1 – RATS checklist

| **Topics** | **Checklist item** | **Reported on page #** |
| --- | --- | --- |
| **Relevance of study question** | Research question explicitly stated | page 7 |
|  | Research question justified and linked to the existing knowledge base (empirical research, theory, policy) | pages 6 and 7 |
| **Appropriateness of qualitative method** | Study design described and justified i.e., why was a particular method (e.g., interviews) chosen? | pages 7 and 15 |
| **Transparency of procedures** | Sampling: Criteria for selecting the study sample justified and explained | pages 7, 8 and 15 |
|  | How recruitment was conducted and by whom | pages 8 and 9 |
|  | Who chose not to participate and why | page 8 |
|  | Method(s) outlined and examples given (e.g., interview questions) | page 9 |
|  | Study group and setting clearly described | page 9 |
|  | End of data collection justified and described | pages 9 and 15 |
|  | Role of researchers | page 10 |
|  | Informed consent process explicitly and clearly detailed | pages 8 and 9 |
|  | Anonymity and confidentiality discussed | pages 8 and 9 |
|  | Ethics approval cited | page 10 |
| **Soundness of interpretive approach** | Analytic approach described in depth and justified | pages 9 and 10 |
|  | Description of the basis on which quotes were chosen, semi-quantification when appropriate, illumination of context and/or meaning, richly detailed | pages 16 to 19 |
|  | Method of reliability check described and justified | Pages 10 and 15 |
|  | Findings are presented with reference to existing theoretical and empirical literature. | pages 16 to 19 |
|  | Strengths and limitations explicitly described and discussed | page 15 |
|  | Evidence of following guidelines (format, word count), detail of methods or additional quotes contained in appendix, written for a health sciences audience | - |
|  | Are red flags present? | - |
